# Opportunities and considerations for using artificial intelligence in bioinformatics education

**DOI:** 10.1093/bioadv/vbaf169

**Published:** 2025-09-01

**Authors:** Stephen R Piccolo, Aparna Nathan, Michelle D Brazas, Manoj Kandpal, Aida T Miró-Herrans, Adam J Kleinschmit, Susan McClatchy, Pertunia Mutheiwana, Dusanka Nikolic, Luciana I Gallo, Rolanda Sunaye Julius, Marta Lloret-Llinares, Nicola Mulder, Danielle Presgraves, Sonal Shewaramani, Jorge Xool-Tamayo, Frédéric J J Chain, Silvia Arantza Sanchez Guerrero

**Affiliations:** Department of Biology, Brigham Young University, Provo, UT, 84602, United States; Department of Biomedical Informatics, Harvard Medical School, Boston, MA, 02115, United States; Adaptive Oncology, Ontario Institute for Cancer Research, Toronto, ON M5G 0A3, Canada; Center for Clinical and Translational Science, The Rockefeller University, New York, NY, 10065, United States; Academic Research Consulting and Services, George A. Smathers Libraries, University of Florida, Gainesville, FL, 32610, United States; Department of Natural and Applied Sciences, University of Dubuque, Dubuque, IA, 52001, United States; The Jackson Laboratory, Bar Harbor, ME, 04609, United States; Computational Biology Division, Department of Integrative Biomedical Sciences, Faculty of Health Sciences, University of Cape Town, Observatory, Cape Town, 7925, South Africa; Wellcome Connecting Science, Wellcome Genome Campus, Hinxton CB10 1SA, United Kingdom; Instituto de Fisiología, Biología Molecular y Neurociencias, CONICET, Departamento de Fisiología y Biología Molecular y Celular, Facultad de Ciencias Exactas y Naturales, Universidad de Buenos Aires, Buenos Aires, C1428EGA, Argentina; Computational Biology Division, Department of Integrative Biomedical Sciences, Faculty of Health Sciences, University of Cape Town, Observatory, Cape Town, 7925, South Africa; EMBL’s European Bioinformatics Institute, Hinxton CB10 1SD, United Kingdom; Computational Biology Division, Department of Integrative Biomedical Sciences, Faculty of Health Sciences, University of Cape Town, Observatory, Cape Town, 7925, South Africa; The Jackson Laboratory, Bar Harbor, ME, 04609, United States; National Microbiology Laboratory Branch, Public Health Agency of Canada, Winnipeg, MB R3E 3R2, Canada; Departamento de Innovación Biomédica, Centro de Investigación Científica y de Educación Superior de Ensenada (CICESE), Ensenada, Baja California, 22860, México; Department of Biological Sciences, University of Massachusetts Lowell, Lowell, MA, 01854, United States; Bachillerato Tecnológico de Educación y Promoción Deportiva (BTED), Plantel Tetla de la Solidaridad, Prolongación República de Brasil s/n 2da secc, Teotlalpan, C.P. 90430, Tetla de la Solidaridad, Tlaxcala, México

## Abstract

Artificial intelligence (AI) tools and techniques are undoubtedly being used in bioinformatics education, reflecting broader trends in education. However, many instructors and learners may be unaware of the full scope of potential uses for these tools within bioinformatics education, as well as effective practices for using them. Building on discussions held at the 6th Global Bioinformatics Education Summit, this perspective article provides insights about ways that AI might be used to generate or adapt instructional content, provide personalized help for learners, and automate assessment and grading. Additionally, we highlight AI skills that are important for bioinformatics learners to develop in order to effectively use AI as a bioinformatics learning tool. We highlight currently available tools in the quickly evolving AI landscape and suggest ways that instructors or learners might use such tools. Furthermore, we discuss key considerations and challenges associated with integrating AI into bioinformatics education, including ethical implications, potential biases, and the need to critically evaluate AI-generated content. Finally, we highlight the need for further research to better understand how AI tools are being used in practice and empower their effective and responsible use in bioinformatics education.

## 1 Introduction

Artificial intelligence (AI) has been used in educational settings for decades (Zawacki-Richter *et al.* 2019). However, recent advances in generative AI (GenAI) and natural language processing have expanded educators’ views about AI’s potential in education. Instructors and learners are experimenting with these tools as a way to develop educational materials, provide personalized support to learners, and equip learners with skills that they will need for the modern workforce. However, many instructors and learners may still be unaware of how these tools can improve their educational experience, especially for domain-specific applications. This review addresses these potential opportunities for bioinformatics education, which we define broadly as efforts to train individuals in concepts and skills necessary to process, analyze, and interpret biological data. In some cases, these efforts aim to help learners with computational or quantitative backgrounds to apply their skills in biology-related contexts. In other cases, the training aim is to help biology-oriented learners expand their skills and leverage the ongoing deluge of biological data. In cases where learners have prior training in biology, computing, and quantitative disciplines, the training aim might be to unite these concepts and skills in interesting and useful ways.

While many bioinformatics educators and practitioners are likely familiar with AI methodologies, many may not fully appreciate their pedagogical role. One goal of this paper is to raise awareness about how AI might enhance the learning and training process. Another goal is to identify available evidence to support the use of AI tools in education and to discuss potential advantages and disadvantages of their use. As often as possible, we connect our observations to bioinformatics specifically; however, we draw heavily from the broader education literature.

In May 2024, we and others gathered in New York City for the 6th Global Bioinformatics Education Summit. In a session titled, “How to use AI in training,” participants discussed their experiences using—or potential to use—diverse types of AI tools for bioinformatics education. Here we highlight four topics related to using AI tools: (i) generating or adapting instructional content, (ii) automating assessment and grading, (iii) providing personalized help to learners, and (iv) teaching learners to use AI. We use *instructor* to refer to any individual who provides bioinformatics training. Such training might occur in formal settings—as in higher-education institutions or secondary schools—or less-formal settings, such as short-form workshops, tutorials, or online courses. Instructors include faculty, staff scientists in academia or industry, postdoctoral researchers, graduate students, freelancers, and other domain experts. We use *learner* to refer to any individual who participates in bioinformatics training. Learners include students matriculated in degree programs, professionals seeking continuing education, researchers expanding their skill sets, and individuals pursuing self-directed learning through online resources or community initiatives. We have structured this article according to the above topics and draw on our summit discussions for insights. We believe these topics are important for bioinformatics educators in diverse settings around the world, as reflected by summit participation from individuals working at academic institutions, industry organizations, nonprofits, or governments on five continents.

## 2 Generating or adapting instructional content

Bioinformatics education requires diverse instructional content, including course outlines, instructional materials (e.g. lecture slides, handouts, and hands-on exercises), coding tutorials, and rubrics. Instructors typically develop these materials themselves, ensuring that the materials align with learning objectives, are grounded in real-world biological problems, and promote active engagement. Ideally, the content follows a logical progression, starting with foundational concepts and advancing to more complex topics and skills. These steps require a significant amount of time and mental effort, which can be amplified when the instructor lacks depth of expertise in the content areas of instruction. Therefore, these processes are ripe for the application of AI tools. In particular, GenAI—including large language models (LLMs)—offer opportunities to accelerate and improve these processes.

As an alternative to creating materials de novo, instructors can write GenAI prompts, requesting help in formulating ideas and pedagogical strategies. Instructors can use GenAI tools as “sounding boards,” providing feedback on approaches they are considering, exploring new perspectives, and helping to verify the correctness of their content. In some cases, GenAI tools can generate new content by adapting existing materials. For example, a hands-on exercise that addresses a particular learning objective might already exist (in a public repository or shared by a colleague); however, the materials might be from a non-biology discipline or from a different biology subdiscipline. Instructors may find GenAI tools helpful in refining those materials and/or contextualizing them. As another example, an instructor could refine existing code snippets by generating line-by-line explanations or characterizing common coding mistakes (MacNeil *et al.* 2022).

In addition to generating static materials, GenAI tools may be useful for interactive learning. For example, an instructor can create a chatbot that provides instructional support to learners. In doing so, the instructor does not directly generate the content but facilitates its creation by training the chatbot on course materials. Chatbots can either be built from scratch or adapted from publicly available frameworks for educators. They can be tailored to a particular content area through custom training or retrieval-augmented generation. In OpenAI’s ecosystem, Custom GPTs offer a way to fine tune GPT models based on specific content. Several Custom GPTs specific to bioinformatics and genomics have been created (Table 1). These chatbots promise to empower learners with diverse skills to understand bioinformatics-related code, analysis workflows, and best practices.

**Table 1.** This table highlights specific tools relevant to bioinformatics education, providing examples of their potential uses for instructors and learners and important considerations when using them.^a^

Category	Example tools	Uses	Cautions
General-purpose LLM chatbots	ChatGPT (OpenAI) Gemini (Google) Claude (Anthropic) Llama (Meta) Many other open-source options (Hugging Face)	Generate content, including course outlines, lesson plans, learning objectives, rubrics, formative assessments, and summative assessments Generate code in response to prompts Explain concepts or code Identify and bridge knowledge gaps Personalize and adapt learning interactively Design or adapt for accessibility Translate from one language to another Automate image or audio description Convert text to speech or speech to text Can be customized, re-trained or augmented for bioinformatics applications	Not specialized for bioinformatics May not be trained on current information Require effective prompts and sometimes iterative prompting Results may not be reproducible due to model stochasticity Results may differ for different versions Often require paid subscriptions or other costs Training may have involved unethical labor and intellectual property practices Learners may rely too heavily on them Generated code may result in logical errors, inefficiencies, or security flaws Service providers may not adequately protect data privacy
Fine-tuned LLM chatbots	Bioinformatics Buddy (https://www.yeschat.ai/gpts-9t55QeNOL4q-Bioinformatics-Buddy) Bioinformatics Manual (https://chatgpt.com/g/g-PHoilMfWI-bioinformaticsmanual) Advanced Bioinformatics and Genomics (https://chatgpt.com/g/g-PHoilMfWI-bioinformaticsmanual)	Support the above uses in a way that is tailored to bioinformatics Facilitate integration with bioinformatics-related data sources Facilitate easier interaction with bioinformatics tool documentation	May lack context of broader concepts May have difficulty extrapolating across disciplines May be updated frequently, potentially requiring repeated adaptation
Specialized LLMs	Tutor (https://chatgpt.com/g/g-0VrB7ieTW-advanced-bioinformatics-and-genomics-tutor) Bioinformatics Expert (https://chatgpt.com/g/g-LcdlLojaA-bioinformatics-expert) AI Photo and Video Generator (Gencraft) Firefly (Adobe) Copilot (Microsoft, GitHub) Xcode extension for GitHub Copilot (GitHub) Windsurf Editor (Codeium)	Generate images or videos for lectures and other educational materials Generate or modify code for activities Act as a pair programmer to personalize learning Provide explanations of code, tools, and commands	Trained models may violate copyright laws May require learners to gain experience with Integrated Development Environments or other specialized tools
Custom LLM-based chatbots	Poe.com (Quora) GPT Builder (OpenAI)	Create custom chatbots	May be time- and effort-intensive

a Each row builds upon the previous one, meaning that the uses listed in earlier rows often apply to tools in later rows, which may introduce additional or specialized functionalities. This list is not exhaustive: multiple versions of these tools may exist and many other tools are available. We do not endorse any specific tool or guarantee improvements in effectiveness, efficiency, or regulatory compliance. Instead, this table serves as a starting point for exploring current capabilities and identifying tools that may support bioinformatics education.

When generating educational content and deciding how to engage with learners, instructors can improve inclusivity and accessibility by delivering content through multiple media, such as written materials, hands-on activities, and discussion (CAST Inc 2025). Summit participants noted that if GenAI tools can reduce some barriers to learning for diverse learners, these tools would address a great need within the bioinformatics community. For example, GenAI can mitigate language biases by generating translated materials for non-native speakers; facilitate inclusive learning environments for remote learners by mimicking group learning (an LLM acts as a group member); cater to different learning styles; and improve learning access by generating text-to-speech or speech-to-text solutions for visually or hearing impaired learners, as reviewed elsewhere (Gibson 2024). However, this benefit is not a guarantee; AI is also known to reflect human biases, amplify disparities, and reinforce accessibility barriers (Quinn 2021, Nadis 2023, Venkit *et al.* 2023, Glickman and Sharot 2025). Inclusive use of AI in bioinformatics education requires thoughtful design and inclusion of individuals from diverse backgrounds into the design and testing of AI-enabled pedagogical tools.

## 3 Providing personalized help to learners

Bioinformatics learners often face obstacles when mastering content and skills, both in traditional teaching contexts and self-directed learning. For example, learners might struggle with understanding complex algorithms, interpreting large datasets, or applying programming skills to biological problems. They may also find it challenging to keep up with rapidly evolving tools and resources. Such obstacles may discourage learners and reduce their self-efficacy. However, in a classroom environment, instructors may have insufficient time to assess each learner’s needs and provide personalized help. In self-directed learning experiences, such as reading books or consuming online material, it is rarely feasible for content creators to provide personalized help. GenAI tools offer opportunities to address these challenges by enabling personalized and adaptive experiences, tailored to individual learners’ needs and paces. By taking advantage of these resources, learners may move closer to the ideal of “self-regulated learning,” which is characterized by greater autonomy, motivation, and intentionality (Garg and Rajendran 2024, Ng *et al.* 2024).

Learners can use LLM chatbots to enhance their understanding of topics they are currently studying or to extend their knowledge to more advanced topics (Yilmaz and Karaoglan Yilmaz 2023). For example, ChatGPT has been shown to effectively respond to queries on bioinformatics-related topics like data mining, genetics, and figure interpretation (Hu *et al.* 2024). The colloquial language used by LLM chatbots allows learners to ask questions in a form that is similar to how they might ask an instructor, and if prompted to do so, the chatbot can respond in ways that are suitable for each learner’s specific needs and skill levels. Chatbots can help learners identify knowledge gaps, create personalized lesson plans, or generate material that helps them prepare for assessments; the latter might include practice questions or hands-on exercises (Ellis and Slade 2023, Mollick and Mollick 2023, Yilmaz and Karaoglan Yilmaz 2023). However, current chatbots may fall short for questions and other interactions that require deep reasoning.

In addition to facilitating conceptual learning, LLM chatbots can help with hands-on skills. For example, chatbots can recommend tools for completing analysis tasks and identify advantages and disadvantages of those tools; in some cases, they may be able to guide learners through the process of using them. For tasks that require programming, chatbots can provide personalized help and increase learners’ self-efficacy (Yilmaz and Karaoglan Yilmaz 2023). For example, ChatGPT has been demonstrated to respond effectively to prompts for generating Python code for basic bioinformatics programming tasks (Piccolo *et al.* 2023). When learners encounter errors, such tools can help with explaining error messages and debugging (Chen *et al.* 2023, Sun *et al.* 2024). These capabilities may also support relatively advanced tasks. For example, plugins such as GitHub Copilot may serve as “pair programmers” to accelerate the processes of understanding, writing, and optimizing code.

To complement learners’ efforts to obtain personalized help from AI tools, instructors can facilitate this process. For example, instructors can guide learners to available AI tools and encourage their effective use, cautioning them to avoid overreliance on the tools. Instructors can recommend high-quality, publicly available chatbots or create custom chatbots that are tailored to a course’s content or learning objectives (Ng *et al.* 2024). To facilitate self-regulation, instructors can encourage or require learners to complete formative exercises without using AI tools—or attempt to generate exercises that AI tools cannot solve—thus enabling the learners to evaluate their own learning. As discussed below, instructors can also teach students best practices for using these tools.

Learners, instructors (and/or content creators), and AI tools each have something to contribute to the learning process. Leveraging the strengths of each can create a more effective and engaging learning environment (Li *et al.* 2024).

## 4 Automating assessment and grading

Assessments play critical roles in educational settings (Dixson and Worrell 2016). Instructors use *formative* assessments to provide feedback during the learning process. Such feedback can help learners identify areas for improvement and help instructors adjust their teaching strategies in response to learners’ progress. *Summative* assessments help instructors evaluate the extent to which learning outcomes have been met; they typically result in a rating or score that quantifies the learner’s performance. In the context of bioinformatics education, assessments might include programming exercises, projects, written reports, and multiple-choice questions. When assessing these contributions, instructors might evaluate written or oral summaries against a rubric, verify that computer code functions properly and follows formatting requirements, or provide feedback on ideas and modes of presentation. However, providing useful feedback can be time consuming and tedious, which may prevent learners from receiving such feedback or receiving it in a timely manner. Creating and delivering summative assessments can be similarly challenging. AI tools have potential to reduce these barriers (González-Calatayud *et al.* 2021, Mollick and Mollick 2023).

With the overarching goal of ensuring that learners receive useful and timely feedback on assessments, we discuss techniques for facilitating this process (Zawacki-Richter *et al.* 2019). In some settings, instructors can use AI tools to customize assessments based on learners’ strengths and weaknesses (e.g. adaptive testing, on-demand chatbots). For programming exercises and other tasks that involve computations, many instructors have access to software tools that automate the process of delivering instructions, feedback, and grades to learners (Messer *et al.* 2023, Piccolo  2025). Machine-learning research and tools within this domain have often focused on assessing code correctness (functionality or methodology). Less research emphasis has been placed on using machine-learning techniques to evaluate whether learners’ code is maintainable, readable, and well documented (Messer *et al.* 2023). We believe the latter skills are important to evaluate because they require deep levels of understanding. Aside from assessing hands-on computing skills, AI techniques can help with assessing higher-order cognitive skills. For example, instructors can deliver open-response assessment items in large courses and use LLM tools to summarize learners’ responses and automate assessment and feedback (Gao *et al.* 2024). Such approaches may enhance the consistency and objectivity of assessments. AI-powered tools may reduce instructors’ reliance on teaching assistants to manually assess learners’ submissions or the use of multiple-choice assessments, which can limit opportunities for nuanced feedback and deeper evaluation of critical thinking skills. Alternatively, AI tools may aid in increasing the quality of multiple-choice distractors, potentially in a data-driven manner based on real learners’ misconceptions.

By using AI tools to save time on tasks that have previously demanded considerable time and energy, instructors may redirect these resources to other tasks (which might also be aided by AI), such as generating practice assessments; meeting one-on-one with learners to assess competencies in more nuanced manners; reviewing assessment instruments and rubrics to improve their alignment with learning outcomes; identifying learners most at risk of performing poorly and helping those learners in the early stages of learning; evaluating their own teaching through summarizing course evaluations; enhancing content (e.g. interlacing dynamic multimedia content with real-time assessment); and identifying and addressing cases of academic dishonesty.

AI tools may also be useful for learners to self-assess. For example, learners can use AI to positively influence their own motivation, increase their engagement with material (e.g. personalized learning plans and content), reduce anxiety, and obtain feedback on written text (Owan *et al.* 2023).

## 5 Teaching learners to use artificial intelligence

As these examples illustrate, AI tools have the potential to transform teaching and learning in bioinformatics. However, as studies conducted in other computing-education settings have found, learners’ baseline use of AI tools does not always promote effective learning (Ghimire and Edwards 2024, Sheese *et al.* 2024). For bioinformatics learners to maximally—and equitably—benefit from AI tools in the classroom, they need to learn how to use these tools effectively. This begins with making AI literacy a pillar of bioinformatics curricula. Literacy is an emancipatory pedagogical concept: it empowers learners to access and share knowledge in an evolving society. Long and Magerko (2020) identified 17 competencies in their consolidated definition of AI literacy across heterogeneous stakeholders. Using this as a foundation, we identify priorities for AI literacy in bioinformatics training: (i) understanding how AI tools work, (ii) recognizing what tasks are well-suited for these tools, (iii) developing good practices to use these tools, and (iv) reflecting on the global impact and ethical costs—intellectual, environmental, labor—of AI tools (Ahmad *et al.* 2023, Crawford 2024).

These learning objectives can be incorporated into bioinformatics training through exercises such as “prompt engineering” (Denny *et al.* 2024). Instructors can provide explanations and examples of how to develop clear prompts and how to iterate and refine prompts given AI tools’ responses and the required bioinformatics task (Sun *et al.* 2024). Training learners to formulate effective prompts for LLMs not only improves their ability to use these AI tools but also requires understanding how the tools work. These exercises also provide opportunities for iteration, error resolution, and self-reflection about their problem-solving strategy and limitations of AI, refining learners’ understanding of when these tools are best deployed. Framing such exercises in bioinformatics contexts can create opportunities to practice core bioinformatics analyses (Shue *et al.* 2023), while also building other important skills for the field, such as navigating uncertainty in the deployment of black-box models (Bearman and Ajjawi 2023) and critical thinking to design and debug code (Styve *et al.* 2024).

Learners should also be introduced to how AI tools’ inherent limitations may introduce inaccuracy or inequity. One hands-on pedagogical strategy is to ask learners to identify potential limitations of the AI tools and design experiments to evaluate the consequences. For example, learners may evaluate an AI tool’s ability to write accurate code in widely used versus less commonly used programming languages; to propose robust analysis steps for multi-ancestry versus European-only datasets; or to write code that can be run in low-resource computing environments. Learners can also investigate the costs of their use of AI tools for bioinformatics analyses. This might include estimating energy and water consumption that results from employing GenAI models (Lannelongue *et al.* 2021, Budennyy *et al.* 2022), identifying potentially inappropriate uses of others’ intellectual property in GenAI responses, or learning about the labor, land, and other social costs of developing these tools (Solaiman *et al.* 2024).

Educating bioinformatics learners on how to use AI can empower beginners and advanced bioinformaticians alike to be informed and responsible users of these tools, while they build an AI skill set essential for the modern workforce and society at large. If implemented thoughtfully, this can even democratize bioinformatics education by giving learners the tools for sustained, catalytic learning beyond what they may have access to at their own institutions (Williams *et al.* 2023).

## 6 Examples

To make these concepts more concrete and illustrate how GenAI could be used in real-world bioinformatics-education contexts, we have provided examples (Additional Data File S1, available as supplementary data at *Bioinformatics Advances* online) for the above categories. In each example, we indicate whether an instructor, a learner, or both might perform the specified task. Where applicable, we provide the GenAI prompt and the model’s response for each example, where relevant. Finally, we provide evaluative comments about the responses. This list is not intended to be comprehensive but rather to highlight some potential use cases. Overall, we conclude that the generated responses could serve as effective starting points for additional refinement.

## 7 Discussion

In this perspective, we provide an overview of the current landscape of AI in bioinformatics education, alongside practical recommendations for further innovation. We acknowledge, however, that these topics do not encompass all potential uses of AI in bioinformatics education and that tools and techniques in these areas are rapidly evolving. Even as newer technologies emerge, opening new possibilities, we believe that certain core considerations will remain critical and relevant.

Notably, the uses of AI in bioinformatics education that we review here are motivated by pedagogical best practices. Rather than replacing the efforts of instructors or learners, these tools can augment their efforts. For example, using AI chatbots for personalized learning does not replace instructors. Human instruction is still important to the learning process, but chatbots provide a means for active learning to continue outside of person-to-person instruction. Additionally, an instructor’s bioinformatics domain expertise remains essential to ensure the quality of AI-generated content; instructors should *not* rely solely on AI tools for tasks such as content creation and ideation (Mollick and Mollick 2023, Yadav 2023).

The emergence of AI tools for education should motivate instructors to learn more about how these tools work and best practices for using them. While it may seem straightforward to use AI tools in educational contexts, our experiences suggest that obtaining high-quality results is not trivial in reality. For example, seemingly small differences in prompts can result in dramatically different outputs. Thus, effective prompt generation is an important skill that requires practice for both instructors and learners. Developing this skill can prevent instructors and learners from feeling frustrated or discouraged when using AI. Effective strategies include providing clear instructions; using short, sequential prompts; providing contextual information; illustrating through examples; and specifying desired output formats (Wang *et al.* 2024).

While AI tools offer valuable support for both instructors and students, they also come with a time investment. If the cost of using these tools outweighs the benefits, their adoption may be impractical for educators. Additionally, we emphasize the need to train instructors in AI-related skills. Although certain tasks may feel intuitive to some, others will require more structured guidance. Beyond crafting effective prompts, instructors may need support in selecting the most appropriate tools for specific tasks; evaluating AI-generated content; helping students interact productively with AI and critically evaluate AI-generated content; and using these technologies in ethically responsible ways. Such training could occur through a range of formats, including conference tutorials, independent workshops, peer interactions, institutional initiatives, and the creation of prompt libraries (Prompt Library 2025).

Building a strong, foundational understanding of AI tools can increase educators’ awareness of the tools’ limitations. AI tools are subject to stochasticity—the same prompt can produce different results—which can lead to variability in the information that learners receive. Instructors should account for this variability in the design of activities and assignments involving AI. GenAI tools mimic a conversational tone without expressing uncertainty, which can lull learners into a false confidence about the tools’ accuracy. Without proper training to navigate the “black-box” nature of these tools, learners may use them blindly, without thinking critically about the outputs, which could undermine the instructor’s learning objectives.

The use of AI tools in education raises ethical concerns, including about environmental impact. Many tools are trained on large corpuses of materials from the Internet but fail to cite those sources in their outputs. Thus, when instructors use GenAI to create course materials, they may unknowingly infringe on others’ intellectual property. Other ethical issues more directly impact learners’ experiences. For example, LLMs are mainly trained on English corpuses and thus produce higher-quality outputs in response to English prompts (Zhang *et al.* 2023), indicating that these tools may disadvantage learners from different language backgrounds. AI tools are inaccessible to many learners who lack Internet access or face socioeconomic barriers (Bulathwela *et al.* 2024). Moreover, commercial AI tools have privacy policies that may leave learners’ personal and educational data vulnerable (Tlili *et al.* 2023). If incorporating AI tools alienates learners in these ways, it ultimately undermines their learning.

Nonetheless, we propose that bioinformatics, an interdisciplinary endeavor, is ideally situated to integrate AI into its training in meaningful ways. This will require thoughtful implementation of free or low-cost tools, trained on diverse corpuses, to serve diverse learners, using AI sandboxes to protect learners’ data. It will require iterative evaluation to ensure that the tools are having the intended impact on core AI literacy and bioinformatics learning outcomes, especially before they are integrated into high-stakes assessments (Hornberger *et al.* 2023).

Researchers and practitioners in the field may find it worthwhile to undergo collective efforts, such as developing high-quality prompts for common bioinformatics educational tasks. As bioinformatics instructors test AI tools in their courses, they should not only reflect on their own experiences but also ask learners for feedback on how the tools impact their learning. For example, do learners find AI-generated assessment feedback valuable? Does questioning a chatbot mimic the experience of questioning an instructor? It is essential for the results of such investigations to be disseminated to the community. There is currently a dearth of literature about applications of AI tools in bioinformatics education; for this review, we drew from literature in both bioinformatics and related fields such as computer science. As AI tools continue to evolve, bioinformatics educators can play a critical role in testing and validating emerging tools and developing best practices and shared resources designed for this specific domain.

## Supplementary Material

vbaf169_Supplementary_Data
