# Supplementary material for: Opportunities and considerations for using artificial intelligence in bioinformatics education
Source: Bioinform Adv. 2025 Sep 1;5(1):vbaf169. doi: 10.1093/bioadv/vbaf169 (PMC12401575; doi:10.1093/bioadv/vbaf169)
Supplement: vbaf169_Supplementary_Data [file vbaf169_supplementary_data.pdf]

Example #1: A BLAST project for beginners

Category: Generating or adapting instructional content

Perspective: Instructor

Prompt:

Generate a programming assignment that requires beginning bioinformaticians to implement a core component of the BLAST alignment algorithm from scratch in the Python programming language. Assume that these learners have a basic capability with Python programming (conditionals, loops, functions, lists, dictionaries, and basic regular expressions) and a basic understanding of biology and bioinformatics, including DNA sequence alignment. Provide a detailed summary that explains the context of the problem, includes learning objectives, and explains how they will be assessed. For the assessment, provide an example solution and at least 3 test cases, along with expected outputs.

Response:

Here's a detailed programming assignment designed for beginning bioinformaticians to implement a simplified version of the **seed-and-extend** phase of the **BLAST (Basic Local Alignment Search Tool)** algorithm. It is structured to match your students' current skills in both **Python programming** and **bioinformatics**.

🧬 Assignment Title:

Implementing a Simplified Seed-and-Extend BLAST Algorithm in Python

---

📘 Context and Overview

BLAST is one of the most widely used tools in bioinformatics, enabling fast comparison of query sequences against databases to find regions of local similarity. One of BLAST's core concepts is **seed-and-extend**: it first identifies short **exact matches (seeds)** between sequences, then **extends** these seeds into longer alignments, scoring them as it goes.

In this assignment, students will implement a simplified version of this strategy:

- **Step 1**: Find exact k-mer matches between a **query** and a **target** DNA sequence.
- **Step 2**: Extend each seed left and right using a simple scoring system to find the best local alignment.

This hands-on implementation will help students understand the core principles of BLAST while practicing problem-solving in Python.

🎯 Learning Objectives

By the end of this assignment, students will be able to:

1. Explain the seed-and-extend strategy used in local sequence alignment.
2. Implement basic string-matching (k-mer hashing) techniques in Python.
3. Apply a simple scoring system to extend seed matches and evaluate alignments.
4. Write modular Python code using functions and control structures.
5. Interpret BLAST-like results, including alignment scores and positions.

📄 Assignment Instructions

✅ Part 1: Input and Setup

Write a function that accepts:

- A query DNA sequence (string of A, C, G, T)
- A target DNA sequence (same format)
- A k-mer size (e.g., `k = 3`)
- A scoring system:
  - Match = +1
  - Mismatch = –1

- No gaps (to simplify)

✅ Part 2: Seed Finding

- Build a dictionary of all **k-mers** in the query sequence with their positions.
- Slide a k-mer window across the target sequence and check for exact matches in the query.

✅ Part 3: Extend the Seeds

- For each seed match, extend **left and right** as long as the score does not drop below zero.
- Report the best alignment: the aligned region, start/end positions in query and target, and the score.

📝 Example Input

```
query = "ACGTCGACG"
target = "GGACGTCGACCCT"
k = 3
```

📋 Assessment Criteria

Criterion	Points
Correct implementation of k-mer seeding	20
Correct extension logic and scoring	25
Modularity and readability of code	15
Handling of edge cases (e.g., no match)	10
Accurate results with test cases	20
Documentation and comments	10

✅ **Example Solution (Simplified)**

```
def find_kmer_seeds(query, target, k):
    query_kmers = {}
    for i in range(len(query) - k + 1):
        kmer = query[i:i + k]
        query_kmers.setdefault(kmer, []).append(i)

    seeds = []
    for j in range(len(target) - k + 1):
        kmer = target[j:j + k]
        if kmer in query_kmers:
            for i in query_kmers[kmer]:
                seeds.append((i, j))  # (query_start, target_start)
    return seeds

def extend_seed(query, target, q_start, t_start, k):
    q_len, t_len = len(query), len(target)
    score = k  # initial match length = k, assuming exact match
    match = 1
    mismatch = -1

    left = 0
    while q_start - left - 1 >= 0 and t_start - left - 1 >= 0:
        if query[q_start - left - 1] == target[t_start - left - 1]:
            score += match
        else:
            score += mismatch
        if score < 0:
            break
        left += 1

    right = 0
    while q_start + k + right < q_len and t_start + k + right <
t_len:
        if query[q_start + k + right] == target[t_start + k + right]:
            score += match
```

```
        else:
            score += mismatch
        if score < 0:
            break
        right += 1

    q_aln_start = q_start - left
    q_aln_end = q_start + k + right
    t_aln_start = t_start - left
    t_aln_end = t_start + k + right

    return {
        "query_aln": query[q_aln_start:q_aln_end],
        "target_aln": target[t_aln_start:t_aln_end],
        "score": score,
        "query_pos": (q_aln_start, q_aln_end),
        "target_pos": (t_aln_start, t_aln_end)
    }

def run_blast(query, target, k):
    seeds = find_kmer_seeds(query, target, k)
    best_result = {"score": -1}
    for q_start, t_start in seeds:
        result = extend_seed(query, target, q_start, t_start, k)
        if result["score"] > best_result["score"]:
            best_result = result
    return best_result
```

🧪 **Test Cases**

🔹 **Test Case 1**

```
run_blast("ACGTCGACG", "GGACGTCGACCCT", 3)
```

✅ Expected Output:

```
{
```

```
    'query_aln': 'ACGTCGACG',
    'target_aln': 'ACGTCGACC',
    'score': 7,
    'query_pos': (0, 9),
    'target_pos': (2, 11)
}
```

🔹 **Test Case 2**

```
run_blast("ATGGC", "CCCCCATGGA", 3)
```

✅ Expected Output:

```
{
    'query_aln': 'ATGG',
    'target_aln': 'ATGG',
    'score': 4,
    'query_pos': (0, 4),
    'target_pos': (5, 9)
}
```

🔹 **Test Case 3**

```
run_blast("TTT", "GGG", 2)
```

✅ Expected Output:

```
{
    'score': -1  # no seed matches found
}
```

📦 **Extension Ideas (Optional)**

- Add gap support.

- Support protein sequences with a BLOSUM matrix.
- Extend in both directions until a **score drop threshold**.
- Return all local alignments above a certain score.

Let me know if you'd like a version of this assignment in Jupyter Notebook format for students or with grading rubrics built in.

Our observations:

- We tested the example solution. The output matches for Test Case 1 and Test Case 3. However, for Test Case 2, the output suggested by the LLM is slightly different from what was generated when executing the code locally. However, the differences were minor.
- The example solution mostly uses techniques that would likely be understandable to a person who has successfully completed an introductory Python course. However, it uses at least one technique (the `setdefault` function) that is relatively advanced and may not be covered in an introductory course.
- The test cases are relatively simple. Requesting more advanced test cases would likely be important.
- Although the example solution contains some basic comments, the example solution could be made more understandable if additional comments were provided.

Example #2: Explaining Python code

Category: Generating or adapting instructional content

Perspective: Instructor

Prompt:

Previously, you generated an example solution for a Python-based BLAST assignment. I want to use the example solution for didactic purposes. Although many of my learners might get their code to produce the expected outputs, they want to see a more effective and/or succinct example to guide their future learning. Give me a different version of the example solution that provides extensive comments. Especially provide clear explanations of relatively complex parts of the code and relatively advanced techniques used.

Response:

Here’s a revised version of the **example BLAST solution**, designed specifically for **didactic clarity**. It includes detailed comments and explanations throughout, with a focus on helping **beginning bioinformaticians** learn good coding habits, understand logic flow, and recognize useful Python idioms.

🧬 Didactic Version: Seed-and-Extend BLAST Implementation (Python)

```
def find_kmer_seeds(query, target, k):
    """
    Step 1: Find exact k-mer matches (seeds) between the query and target sequences.

    We create a dictionary mapping each k-mer in the query to a list of its positions.
    Then we slide a k-mer window over the target and check for matches in that dictionary.

    Returns a list of tuples: (query_start, target_start)
    """
    query_kmers = {}  # Dictionary to store query k-mers and their positions

    # Build index of all k-mers in the query
    for i in range(len(query) - k + 1):
        kmer = query[i:i + k]
        if kmer not in query_kmers:
            query_kmers[kmer] = []
        query_kmers[kmer].append(i)  # Store all occurrences of the k-mer

    seeds = []

    # Slide a k-mer window across the target and check for matches
    for j in range(len(target) - k + 1):
```

```
        kmer = target[j:j + k]
        if kmer in query_kmers:
            # For each matching k-mer, add all positions it occurs in
the query
            for query_pos in query_kmers[kmer]:
                seeds.append((query_pos, j))

    return seeds


def extend_seed(query, target, q_start, t_start, k):
    """
    Step 2: Extend a matching seed in both directions using a simple
scoring scheme.

    Match = +1
    Mismatch = -1
    No gaps are allowed (gap penalty is not implemented for
simplicity).

    We extend outward from the seed until the alignment score drops
below 0.
    This mimics the local alignment heuristic of BLAST.

    Returns a dictionary containing the alignment strings, score, and
positions.
    """
    match_score = 1
    mismatch_penalty = -1
    score = k * match_score  # Initial score for the seed itself
(assumed to be an exact match)

    q_len, t_len = len(query), len(target)

    # Initialize how far we can extend left and right
    left = 0
    right = 0
```

```
    # -------- Left Extension --------
    while (q_start - left - 1 >= 0) and (t_start - left - 1 >= 0):
        # Get the next character to compare
        q_char = query[q_start - left - 1]
        t_char = target[t_start - left - 1]

        # Update the score based on match/mismatch
        if q_char == t_char:
            score += match_score
        else:
            score += mismatch_penalty

        # Stop extending if the score drops below 0
        if score < 0:
            break

        left += 1

    # -------- Right Extension --------
    while (q_start + k + right < q_len) and (t_start + k + right <
t_len):
        q_char = query[q_start + k + right]
        t_char = target[t_start + k + right]

        if q_char == t_char:
            score += match_score
        else:
            score += mismatch_penalty

        if score < 0:
            break

        right += 1

    # Calculate final alignment bounds
    q_aln_start = q_start - left
    q_aln_end = q_start + k + right
    t_aln_start = t_start - left
```

```
    t_aln_end = t_start + k + right

    return {
        "query_aln": query[q_aln_start:q_aln_end],
        "target_aln": target[t_aln_start:t_aln_end],
        "score": score,
        "query_pos": (q_aln_start, q_aln_end),
        "target_pos": (t_aln_start, t_aln_end)
    }


def run_blast(query, target, k):
    """
    Main BLAST-like function to run the full seed-and-extend process.

    1. Find all matching seeds.
    2. For each seed, attempt extension.
    3. Keep track of the best-scoring alignment found.

    Returns a dictionary representing the best alignment.
    """
    seeds = find_kmer_seeds(query, target, k)

    best_result = {
        "score": -1,  # Start with a dummy score lower than any
possible match
    }

    for q_start, t_start in seeds:
        result = extend_seed(query, target, q_start, t_start, k)

        if result["score"] > best_result["score"]:
            best_result = result  # Replace with better alignment

    return best_result
```

✅ Key Teaching Highlights in the Code

Section	What It Teaches
`find_kmer_seeds`	Efficient string indexing with dictionaries, avoiding brute-force search
`extend_seed`	Step-by-step logic for local alignment; control flow with break conditions
`run_blast`	Looping with conditionals to find maxima; encapsulation and modularity
Comments & naming	Clarity in naming variables (`q_start`, `t_start`, etc.) for better traceability

---

🧪 Test Case Example (Recap)

```
query = "ACGTCGACG"
target = "GGACGTCGACCCT"
k = 3

result = run_blast(query, target, k)

print(result)
```

✅ Expected Output:

```
{
    'query_aln': 'ACGTCGACG',
    'target_aln': 'ACGTCGACC',
    'score': 7,
    'query_pos': (0, 9),
    'target_pos': (2, 11)
}
```

Let me know if you'd like:

- A **visual diagram** to pair with the seed/extend logic,
- A **Jupyter Notebook version** for interactive student learning,
- Or a **formative quiz** to check understanding after they review this example.

Our observations:

- This example illustrates that an LLM can remember the context of previous prompts and responses and generate follow-up responses.
- Most of the comments focus on the high-level workflow of the code. Less emphasis is placed on details about programming logic or constructs used.

Example #3: Translate a programming exercise from one (human) language to another

Category: Generating or adapting instructional content

Perspective: Instructor or learner

Prompt:

Below is a Python programming exercise designed for novice bioinformaticians. Translate the descriptions from English to Portuguese.

Below are the average weights of 5 types of small mammals:

- Squirrel: 1-1.5 lbs
- Ferret: 2-4 lbs
- Rabbit: 5-7 lbs
- Cat: 8-10 lbs
- Small dog: 11-20 lbs

Here is a list of the weights of some examples of the above mentioned animals.

animalWeights = [3.5, 15, 8.3, 22, 1.2, 4, 6, 17.5, 4.5]

Write a function called `findAnimalsByWeight` that accepts a list such as `animalWeights`, checks each weight in the list, returns a list that contains the name of the animal that most likely corresponds to each weight. For example, an animal that weighs 3.5 lbs. is probably a ferret, and an animal that weights 15 lbs. is probably a small dog, etc.

If the weight does not fit into any of the ranges listed above, return `None`.

(It might be useful to create a second function that accepts a given weight and prints what animal the weight corresponds to.)

Response:

Here is the translated version in Portuguese of the exercise description for novice bioinformaticians:

Abaixo estão os pesos médios de 5 tipos de pequenos mamíferos:
Esquilo: 1–1.5 libras
Furão: 2–4 libras
Coelho: 5–7 libras
Gato: 8–10 libras
Cachorro pequeno: 11–20 libras

Aqui está uma lista com os pesos de alguns exemplos dos animais mencionados acima:

```
animalWeights = [3.5, 15, 8.3, 22, 1.2, 4, 6, 17.5, 4.5]
```

Escreva uma função chamada `findAnimalsByWeight` que aceita uma lista como `animalWeights`, verifica cada peso na lista e retorna uma nova lista que contém o nome do animal que provavelmente corresponde a cada peso.

Por exemplo, um animal que pesa 3.5 libras provavelmente é um furão, e um animal que pesa 15 libras provavelmente é um cachorro pequeno, etc.

Se o peso não se encaixar em nenhum dos intervalos listados acima, retorne `None`.

(Pode ser útil criar uma segunda função que aceita um peso específico e imprime qual animal corresponde a esse peso.)

Let me know if you'd like the solution or further guidance also translated!

Our observations:

- A native speaker of Brazilian Portuguese (who also speaks and writes English proficiently) reviewed the response and provided these comments:
    - The translation "looks fine to me."
    - In many non-English speaking settings, the decimal point is actually a comma. For a Portuguese-speaking audience, "1.5" should be "1,5".
- The English example used pounds (lbs.) as a weight measurement commonly used in the USA. However, this weight system is not used in Portuguese-speaking countries, so it would have been better if the translation were specified in kilograms. This could be requested in the prompt.
- The formatting was lost when the text was copied into the ChatGPT interface. Thus, there was no formatting in the output that ChatGPT produced. It might have been better to take a screenshot of the exercise and paste that into ChatGPT. Or it might work to request that ChatGPT format the output, though the formatting may not match the original formatting.
- ChatGPT translated the descriptions but not the variable names. This depends on the prompter's preference. (We performed a subsequent test and found that it was capable of translating the variable names as well.)

Example #4: Performing a differential-expression analysis

Category: Providing personalized help to learners

Perspective: Learner

Prompt:

I am a bioinformatics learner. I am learning to use the *limma* package from Bioconductor. The user guide is stored in this PDF file: https://bioconductor.org/packages/release/bioc/vignettes/limma/inst/doc/usersguide.pdf. I have a matrix with normalized expression values. The first four columns have expression values for biological replicates that are controls. The next four columns have expression values for biological replications that have been transfected with an shRNA. Now I want to perform a differential expression. Walk me through in a simple way how to perform this analysis and interpret the results.

Response:

Great! You're in a strong place — using **limma** is one of the most powerful and accessible ways to perform **differential expression analysis** on normalized expression data in R. Since you're working with a matrix of already normalized values and two experimental groups (control and shRNA-treated), you can **skip normalization steps** and go straight into **model fitting and testing**.

🧬 Step-by-Step: Differential Expression with limma

Let's assume your normalized expression matrix is called `exprs`, with genes as rows and samples as columns:

```
# Dimensions: rows = genes, columns = samples
dim(exprs)
# [1] 20000    8  # e.g., 20,000 genes × 8 samples
```

Your columns:

- Columns 1–4 = **control**
- Columns 5–8 = **shRNA-treated**

✅ Step 1: Load limma and define sample groups

```
library(limma)

# Define the group labels for your samples
group <- factor(c("control", "control", "control", "control",
                  "shRNA", "shRNA", "shRNA", "shRNA"))
```

✅ Step 2: Create the design matrix

This encodes your experimental setup. We're testing if expression differs between control and shRNA.

```
design <- model.matrix(~ group)
colnames(design)
# [1] "(Intercept)" "groupshRNA"
```

- The second column (`groupshRNA`) will test for the **difference between shRNA and control**.

✅ Step 3: Fit the linear model to your data

```
fit <- lmFit(exprs, design)
```

✅ Step 4: Apply empirical Bayes moderation

```
fit2 <- eBayes(fit)
```

This step improves statistical stability by borrowing information across genes (a core benefit of limma).

✅ Step 5: Extract top differentially expressed genes

```
topTable(fit2, coef = "groupshRNA")
```

- This gives you a ranked table of DE genes based on adjusted p-values.
- Columns you'll see include:
  - `logFC`: log2 fold change (positive = higher in shRNA)
  - `AveExpr`: average expression

- `t`: moderated t-statistic
- `P.Value`: raw p-value
- `adj.P.Val`: FDR-adjusted p-value (Benjamini-Hochberg)
- `B`: log-odds of differential expression

🔍 Interpretation

- **logFC > 0**: gene is upregulated in shRNA-treated samples vs. control
- **logFC < 0**: gene is downregulated
- **adj.P.Val < 0.05**: gene is significantly differentially expressed after correcting for multiple testing
- **B**: a higher B-statistic means higher confidence the gene is truly differentially expressed

---

✅ Optional: Visualize results

Volcano plot:

```
volcanoplot(fit2, coef = "groupshRNA", highlight = 10, names = rownames(exprs))
```

MA plot:

```
plotMA(fit2, coef = "groupshRNA")
```

🧠 Summary

1. **Group definition**: identify sample labels.
2. **Model design**: encode your experiment.
3. **Fit the model**: use `lmFit()` and `eBayes()`.
4. **Extract results**: use `topTable()` to interpret DEGs.
5. **Adjust for multiple testing** using FDR.

Let me know if you'd like to include annotation (e.g. gene symbols), or filter based on specific thresholds!

Our observations:

- It is unclear whether ChatGPT actually parsed through the PDF file or used its internal knowledge about how *limma* works.
- The interpretation portion was helpful, but it did not describe all of the outputs (e.g., t statistic, adjusted p-value), which may require clarification for someone who is not well versed in statistics.
- The concept of fold changes was not clarified. It's important to understand this concept and how the information might be used to filter the results. It would be helpful to explain how these values are calculated (a value of 2.0 means that expression levels are 2 times "higher" in the treated group based on comparing the means).
- Additional clarification would likely be necessary for less-experienced learners.
- The output stops short at explaining some details that might prove to be important for interpretation. For example, the `topTable` function only shows the "top 10" genes by default. Additionally, for the optional visualizations, it isn't explained how to interpret the graphs, in particular the volcano plot. These limitations may inadvertently mislead the learner to think that there are only 10 differentially expressed genes.

Example #5: Explain statistical concepts related to differential-expression testing

Category: Providing personalized help to learners

Perspective: Learner

Prompt:

I want to better understand two aspects about differential expression analyses.

The first is multiple-testing correction. I understand that it gives me an adjusted p-value. But I am not sure I understand why this is necessary. Why can I not just use the regular p-value and the traditional 0.05 threshold? What does FDR mean?

The second is fold change. What does this tell me that is different from what a p-value or FDR tells me? How do I use it when evaluating a list of differentially expressed genes?

Explain these concepts at a basic level. I am just learning how statistics work, especially in the context of transcriptomic data.

Response:

You're asking excellent, foundational questions — and understanding these two concepts is **key to doing meaningful transcriptomic analysis**. Let's break them down simply and clearly.

🧪 Part 1: Why Use Multiple-Testing Correction? What Is FDR?

🔍 The problem with many p-values

In a typical differential expression analysis, you're testing **thousands of genes** (often 20,000+) to see if each one is expressed differently between two conditions.

If you used a regular **p-value threshold of 0.05**, you'd expect:

- **5% of all genes** to be marked "significant" **just by random chance**, even if there's no real difference.

So if you're testing 20,000 genes:

- **0.05 × 20,000 = 1,000 genes** might be "significant" **just due to noise**.

✅ Enter multiple-testing correction

This corrects for the fact that you're doing lots of tests and helps you control the number of **false positives**.

📘 What is FDR (False Discovery Rate)?

FDR is a more intuitive way of handling this.

- The **FDR-adjusted p-value** (from methods like Benjamini-Hochberg) tells you:

  "If I call this gene significant, what’s the chance I’m wrong?"

- So, if an adjusted p-value is **0.01**, you’re saying:

  "I’m willing to accept a 1% chance that this gene is a false positive."

FDR helps you make **more trustworthy discoveries**.

🚫 Why not just use the raw p-value?

Because in big datasets, **too many false positives** will sneak in. You might waste time or resources chasing genes that aren’t truly differentially expressed.

🔬 Part 2: What Does Fold Change Tell Me? How Is It Different From P-values?

🔍 Fold change (FC) shows how big the effect is.

- It tells you how **much** a gene’s expression changes between two groups.
- Often shown as **log2 fold change**:
  - `logFC = 1` → 2× higher in treated group
  - `logFC = -2` → 4× lower in treated group

So while a **p-value tells you if the change is statistically reliable**, a **fold change tells you how biologically meaningful the change is**.

✅ Why you need both

Let's say:

- **Gene A** has a small fold change (1.2×) but very low p-value → statistically strong, but maybe not biologically relevant.
- **Gene B** has a large fold change (6×) but high p-value → maybe interesting, but data is too noisy to trust it.

The **best hits** are:

- Genes with **both low FDR (e.g. < 0.05)** and **high absolute log fold change (e.g. > 1)**

🧠 How to use fold change in practice

When reviewing DE genes:

- **Filter or rank** by both `adj.P.Val` (FDR) and `|logFC|`

For example:

```
subset(results, adj.P.Val < 0.05 & abs(logFC) > 1)
```

This gives you genes that are:

- **Statistically reliable**
- And **biologically meaningful**

✅ Summary

Concept	What It Tells You	Why It Matters
**P-value**	How likely a gene's difference is by chance	Too many false positives when done 20k+ times

**FDR**	Adjusted p-value: chance of false discovery	Controls for multiple testing
**Fold Change**	How much expression changes between groups	Shows biological impact, not just statistical noise

---

Let me know if you'd like an analogy or a real-life example to solidify this further!

Our observations:

- The summary effectively explains these fundamental concepts for the most part. However, more clarity would be useful (see observations from Example #4).
- Follow-up questions might include:
  - Asking what a false positive means more broadly.
  - What a typical FDR threshold is in the real world and how such a threshold is determined.
  - What a typical log FC threshold is in the real world and how such a threshold is determined.

Example #6: Evaluating students' open-ended responses to a biostatistics question

Category: Automating assessment and grading

Perspective: Instructor

Prompt:

For an introductory biostatistics course at the undergraduate level, the instructor describes a scenario in which a two-sample t test is used. In this scenario, it is most appropriate to use a one-sided test, but the instructor has not yet revealed this to the learners. The instructor asks the learners to consider the information they have been given and answer the question, "Would you use a one-sided or two-sided test and why?" They are given the rubric, "You will be graded on whether you provide the correct answer as well as appropriate justification for your response." Evaluate the following responses, award a letter grade (A-E scale), and provide a short justification for why you awarded these grades. Use an encouraging yet rigorous tone.

Response 1: "I would use a one-sided test."

Response 2: "I would use a one-sided test because the research question implies we have a specific directional hypothesis about which group will have the higher mean. The context suggests we're testing whether one treatment is better than another, not just whether they're different. A one-sided test is more powerful when we have theoretical justification for expecting a difference in a particular direction, and it appears we do based on the scenario described. Using a two-sided test would be unnecessarily conservative here and reduce our ability to detect a real effect."

Response 3: "I would use a two-sided test because even though the scenario suggests we might expect a difference in one direction, we should generally default to two-sided tests unless we have very strong prior evidence. Two-sided tests are more conservative and protect against the possibility that the effect could go in the opposite direction than expected. It's better to be cautious in hypothesis testing."

Response 4: "I think a one-sided test would be appropriate here because it seems like we're expecting the difference to go in a specific direction based on what was described. One-sided tests are used when you have a directional hypothesis, and this appears to be the case. However, I would want to make sure that the research question really supports testing in only one direction before making this decision final."

Response 5: "I would use a two-sided test."

Response:

Here is an evaluation of each response using an A–E letter grade scale, based on whether the answer is correct and includes appropriate justification, in alignment with the instructor's rubric. The tone is constructive and encourages learning while being academically honest.

🟡 Response 1 – Grade: D
You chose the correct type of test, which is a good start. However, to earn full credit, you need to explain your reasoning. It's important to show not just what you chose, but why. In statistical analysis, your justification helps demonstrate that you understand the concepts, not just the answer.

✅ Response 2 – Grade: A
Excellent work! You selected the correct test and provided a clear, well-reasoned explanation. You explained the importance of having a directional hypothesis, and you correctly identified that a one-sided test is more powerful when that direction is justified. You also thoughtfully addressed why a two-sided test would be unnecessarily conservative here. This is a great example of analytical thinking in biostatistics.

🟠 Response 3 – Grade: B
You made a well-reasoned case for choosing a two-sided test, even though the correct answer for this scenario was a one-sided test. What you wrote shows a solid grasp of statistical caution and best practices, especially the idea that we should be careful when assuming directionality. Although you missed the subtle hint that this scenario supported a directional hypothesis, your justification reflects thoughtful reasoning, which is valuable.

✅ Response 4 – Grade: A-
Very good response! You correctly leaned toward a one-sided test and explained that it fits when there's a clear expectation of direction. I also appreciate your thoughtful note about making sure the research question really supports that direction — that's exactly the kind of critical thinking that's important in data analysis. The only reason this isn't a full A is that your answer was a little cautious where the scenario did provide enough information. Still, this is a strong and well-supported response.

🔴 Response 5 – Grade: E
In this case, you chose the incorrect test and didn't include any explanation for your choice. Remember, in biostatistics, we need to justify our decisions so others (and you!) can understand why a certain method is appropriate. This is a learning opportunity — always include your reasoning, and pay close attention to whether the research question hints at a directional hypothesis.

Let me know if you'd like these bundled into grading comments for an assignment or LMS system!

Our observations:

- To avoid violating student privacy, we generated the student responses using the Claude Sonnet 4 LLM rather than using actual student responses.
- Initially, the responses were directed at the instructor. We asked ChatGPT to rephrase the responses, directed at the students.
- The responses are remarkably well done and use an appropriate tone.
- We could have provided a more detailed rubric, which likely would have led to even better responses.

Example #7: Evaluate whether a learner's code is readable and well documented

Category: Automating assessment and grading

Perspective: Instructor

Prompt:

Below is a prompt from a Python programming problem that I give to learners in an introductory bioinformatics course.

> DNA sequences differ between individuals. Thus, although there is a "reference" human genome, your personal genome differs from the reference in many places. Some locations of DNA have frequent variation within a population. In this problem, you will be provided with short DNA sequences representing the reference genome and another sequence representing an individual person's DNA. Your goal is to find the positions that differ between the reference sequence and the individual's sequence.
>
> You should create a function called compareSequences. It should accept two lists. Both of these lists will contain multiple strings. The length of the two lists will be the same. Each string in the lists will contain "A", "C", "G", or "T", representing nucleotides. The first list will represent a reference DNA sequence. The second list will represent the DNA sequence of a non-reference individual.
>
> Your function should return a list. Elements of this list should be the indices (positions) of each nucleotide in which the two sequences differ from each other. For example, if the reference allele at position 6 were a "T" and the individual had an "A", you would include 6 in the list that you return.

I want to assess the extent to which the learners' code is readable and well documented. Below is a detailed rubric.

> Readability (50 points)
>
> 5 - Excellent (45-50 points)
>
> - Variable names are clear and descriptive (e.g., total_score, user_input)
> - Code follows consistent indentation (4 spaces)
> - Logical flow that's easy to follow from top to bottom
> - Proper spacing around operators and after commas
> - Uses appropriate Python syntax and structure

4 - Good (40-44 points)

- Most variable names are clear and descriptive
- Consistent indentation with minimal issues
- Generally easy to follow
- Good use of spacing and Python syntax

3 - Satisfactory (35-39 points)

- Some variable names are clear, others could be improved
- Mostly consistent indentation
- Code is understandable but requires some effort
- Basic Python syntax is correct

2 - Developing (30-34 points)

- Several unclear variable names (e.g., x, temp, data1)
- Some indentation inconsistencies
- Code is somewhat difficult to follow
- Syntax is correct but not well-formatted

1 - Needs Improvement (0-29 points)

- Most variables have unclear or meaningless names
- Inconsistent or incorrect indentation
- Code is difficult to understand
- Poor formatting makes it hard to read

Documentation (50 points)

5 - Excellent (45-50 points)

- Includes a brief comment explaining the overall purpose of the code snippet
- Key lines or complex logic have helpful inline comments
- Comments are concise and add value
- No redundant or obvious comments

4 - Good (40-44 points)

- Has either a purpose comment or good inline comments
- Most comments are helpful and clear
- Minimal redundant commenting
- Documentation aids understanding

3 - Satisfactory (35-39 points)

- Some comments present that help explain the code
- Comments are generally accurate but may be basic
- Could benefit from additional explanation in key areas

2 - Developing (30-34 points)

- Minimal commenting that provides limited help
- Comments may be unclear or too obvious
- Missing documentation where it would be helpful

1 - Needs Improvement (0-29 points)

- No meaningful comments or documentation
- Any comments present are confusing or incorrect
- Code purpose and logic are unclear without explanation

Overall Grade Calculation

Readability Score: _____ / 50 Documentation Score: _____ / 50 Total Score: _____ / 100

Below are solutions from three different students. Generate two paragraphs for each solution that describe how well they met the Readability and Documentation requirements. Use an encouraging tone and provide feedback on what they could have done better. Also give them a point score for each criterion, as well as an overall score.

Solution #1:

```
def compareSequences(ref, ind):
    # Find differences between DNA sequences
    diff = []
    for i in range(len(ref)):
        if ref[i] != ind[i]:
            diff.append(i)
    return diff
```

Solution #2:

```
def compareSequences(a, b):
    x = []
    for i in range(len(a)):
        if a[i] != b[i]:
            x.append(i)
    return x
```

Solution #3:

```
def compareSequences(reference_sequence, individual_sequence):
    """
    Compare two DNA sequences and find positions where nucleotides differ.

    Args:
        reference_sequence: List of strings representing reference DNA
        individual_sequence: List of strings representing individual's DNA

    Returns:
        List of integers representing positions where sequences differ
    """
    different_positions = []

    # Compare each nucleotide position across both sequences
    for position in range(len(reference_sequence)):
        if reference_sequence[position] != individual_sequence[position]:
            different_positions.append(position)


    return different_positions
```

Response:

Here's a detailed evaluation for each solution using the provided rubric:

✅ **Solution #1**

```
def compareSequences(ref, ind):
    # Find differences between DNA sequences
    diff = []
    for i in range(len(ref)):
        if ref[i] != ind[i]:
            diff.append(i)
    return diff
```

Readability (Score: 43 / 50)

This solution is clean, logically structured, and easy to read. The code follows consistent indentation and uses appropriate spacing around operators and within the loop. The flow from input to output is straightforward. Variable names like `ref` and `ind` are somewhat descriptive

but could be improved for clarity — for example, changing them to `reference_sequence` and `individual_sequence` would make the function more self-documenting. The list name `diff` is appropriate for the context but might benefit from a more specific name like `differences` or `mismatch_positions`.

Documentation (Score: 40 / 50)

The function includes a brief comment that explains its general purpose, which is helpful. However, the comment is high-level and does not explain the internal logic or rationale behind the steps taken. Adding inline comments to clarify the comparison logic and the purpose of the loop would enhance the reader's understanding, especially for beginners. Also, including a docstring would bring the documentation to a more professional level.

Total Score: 83 / 100

✅ **Solution #2**

```
def compareSequences(a, b):
    x = []
    for i in range(len(a)):
        if a[i] != b[i]:
            x.append(i)
    return x
```

Readability (Score: 32 / 50)

This solution works correctly, but its readability suffers due to non-descriptive variable names. Variables `a`, `b`, and `x` do not convey any biological or functional context, which makes it difficult to quickly understand what the code is doing. Despite this, the indentation is consistent, and the logic is simple enough to follow. Improving variable names alone would substantially elevate the quality of the solution.

Documentation (Score: 30 / 50)

There are no comments or docstrings in this solution, which makes it harder for others to understand the purpose and logic of the function — especially in an educational context. Including even a basic explanation of what the function does and what the variables represent would be beneficial. As it stands, learners might find this code a bit cryptic without additional guidance.

Total Score: 62 / 100

✅ **Solution #3**

```
def compareSequences(reference_sequence, individual_sequence):
    """
    Compare two DNA sequences and find positions where nucleotides
differ.

    Args:
        reference_sequence: List of strings representing reference
DNA
        individual_sequence: List of strings representing
individual's DNA

    Returns:
        List of integers representing positions where sequences
differ
    """
    different_positions = []

    # Compare each nucleotide position across both sequences
    for position in range(len(reference_sequence)):
        if reference_sequence[position] !=
individual_sequence[position]:
            different_positions.append(position)

    return different_positions
```

Readability (Score: 50 / 50)

This is an excellent example of clear and maintainable code. The variable names are precise and contextually relevant to bioinformatics, making the function self-explanatory. Indentation, spacing, and logical flow are all consistently excellent. The list name `different_positions` is also descriptive and well-aligned with the function's purpose. Overall, it's a textbook example of readable code.

Documentation (Score: 50 / 50)

The documentation is exemplary. The function includes a well-written docstring that clearly explains what the function does, what each parameter represents, and what the function returns. There is also an inline comment inside the loop that concisely explains the logic of comparing nucleotides. This level of documentation is especially helpful in an educational setting and serves as a strong model for peers.

Total Score: 100 / 100

Let me know if you'd like this rubric applied to additional student solutions or if you'd like a markdown version of this rubric to share with your class!

Our observations:

- We generated the rubric and example solutions using the Claude Sonnet 4 LLM rather than using actual student responses.
- This programming exercise can be completed with only a few lines of code. More complex problems would help to better evaluate ChatGPT's ability to evaluate code solutions based on the rubric. However, it did an impressive job.

Example #8: An exercise for evaluating AI-generated content

Category: Teaching learners to use artificial intelligence

Perspective: Instructor

Note:

This exercise focuses on asking students to evaluate AI-generated content but does not use AI to generate the exercise (unlike the previous examples).

Context:

In a bioinformatics course, an instructor seeks to teach skills in evaluating AI-generated context. Specifically, the instructor wishes to emphasize that the wording of a prompt can influence the quality of responses from a language model.

Instructions for learners:

This assignment is an opportunity to develop critical thinking skills when working with an AI chatbot (like ChatGPT). Suppose you are analyzing genomic sequences and want help from a chatbot to count the frequency of each nucleotide in a given DNA sequence. Please do the following.

A. Write three prompts that are different from each other. For example, you could change the level of detail about the task that needs to be performed, the amount of background information you provide about the task, the tone you use, the format of the request (for example, bullet points versus paragraph), and/or the intended audience (for example, beginner vs. experienced learner).
B. Write three different prompts that ask the chatbot to help with this task. These prompts should vary in clarity and level of detail, as outlined below. Be sure to indicate your experience level as a bioinformatician as part of the prompts.

C. For each prompt:

    a. Record your full prompt.

    b. Record the chatbot's response.

    c. Attempt to implement the advice provided by the chatbot.

    d. Write a 75-100 word reflection about the quality of the response.

D. For the prompt that you believe performed best, do the following:

    a. Refine the prompt based on what you think might lead to an even more helpful response.

    b. Write a 75-100 word reflection about the quality of the new response and how it changed in comparison to the first response.

E. Write a 150-200 word reflection about this exercise overall, including what you interpret about the effects of varying the different factors that you changed.

Our observations:

- This exercise could be applied to a wide variety of bioinformatic tasks.
